# The Role of Physicians’ Factors in Underdiagnosis of Celiac Disease in the Eastern Province of Saudi Arabia

**DOI:** 10.7759/cureus.44690

**Published:** 2023-09-04

**Authors:** Abdul Sattar Khan, Baqer M Albaqshi, Ali M Alismael, Abdullah H Bohamad, Ahmed A Almutawah, Ali H Alabdellah, Alhwraa S Almajed, Abdullah S Almajed, Adnan S Almajed

**Affiliations:** 1 Family and Community Medicine Department, King Faisal University, Al-Ahsa, SAU; 2 Critical Care Medicine, Dammam Medical Complex, Dammam, SAU

**Keywords:** saudi arabia, knowledge, attitudes, physicians, celiac disease

## Abstract

Background

Celiac disease is an autoimmune disorder triggered by gluten and related prolamines, which can cause a variety of symptoms and complications if left untreated. Despite being a common lifelong disorder, it often goes undiagnosed for a long time, leading to negative impacts on patients' health and quality of life. The diagnosis of celiac disease requires the presence of celiac-specific autoantibodies and distinctive histological changes in the small intestinal mucosa. Lack of disease knowledge among healthcare professionals and patients' adherence to gluten-free diets may contribute to diagnostic delays.

Objectives

This study aims to assess the reasons for celiac disease underdiagnosis and identify the functional deficiencies of healthcare professionals in the diagnosis and treatment of celiac disease, particularly in the Saudi population.

Materials and methods

A cross-sectional, questionnaire-based study was conducted among physicians in the Eastern Province of Saudi Arabia during the year 2023, between May and July. Participants were asked to complete an online self-administered questionnaire that included questions about their demographic characteristics, professional experience, and knowledge and attitudes toward celiac disease. The study recruited gastroenterologists, gastroenterology fellows, internal and family medicine specialists, residents, and general practitioners working in private or public health centers in various cities of the Eastern Province.

Results

The data were collected from 180 physicians who fulfilled the inclusion criteria of the study, with most participants aged under 30 years and predominantly male. Family medicine and general practitioners were the most represented specialties. While 49.4% of physicians knew that adult celiac disease was rare, only 19.4% frequently recommended celiac disease serology to their patients. In terms of risk, most physicians knew that adult celiac disease was a moderately severe and disabling disease, but only 24.4% thought that the cancer risk in patients with celiac disease was moderate. About 75.6% of physicians had an overall poor knowledge level regarding celiac disease, with gastroenterologists and internal medicine specialists demonstrating better knowledge compared to other specialties (P = 0.001).

Conclusion

The study found that a majority of physicians in the Eastern Province of Saudi Arabia had poor knowledge about celiac disease. This lack of knowledge could have implications for patient care, as it could lead to delays in diagnosis, inadequate treatment, and increased risk of complications.

## Introduction

Celiac disease (CD) is a chronic autoimmune disorder triggered by gluten and related prolamines in genetically predisposed individuals. CD is more common among family members of affected individuals due to its genetic background and is associated with several other conditions, including autoimmune thyroid disease, type-1 diabetes, IgA deficiency, and certain chromosomal abnormalities such as Down syndrome, Turner syndrome, and Williams syndrome. Its clinical manifestations are extremely varied and can be intestinal, extra-intestinal, or even asymptomatic [1]. Growth failure, weight loss, diarrhea, and nutrient deficiencies are among the typical symptoms of the condition. In addition, anemia from iron or folic acid deficiency, hypocalcemia, and osteoporosis may follow [2].

Previously, it was believed that CD typically manifested as a disorder in childhood, but it is now widely acknowledged that symptoms can appear at any age. According to several surveys, the peak age of symptom onset is in the fifth or sixth decade of life [3]. Even though CD is one of the most prevalent lifelong disorders, it often goes undiagnosed for a long time in many patients [4]. Some regions experience diagnostic delays of over ten years, which may have a significant negative impact on patient's health and quality of life [5].

It is obvious that CD detection needs to be improved. Untreated celiac disease is linked to several complications, the most serious of which is malignant lymphoma or small intestine cancer [6]. The presence of celiac-specific autoantibodies and distinctive histological changes in the small intestinal mucosa is required for a definitive diagnosis of celiac disease, which is suspected based on the clinical presentation [7]. An increasing incidence of diagnoses from 1950 to 2001 is shown by an analysis of celiac disease diagnoses in Olmsted County (Minnesota), but there is still a significant underdiagnosis across the country. Why celiac disease is not detected earlier and more frequently is a question that has not yet been addressed. Only 11% of the 2,440 patients surveyed reported having celiac disease diagnosed by a primary care physician (8% confirmed by biopsy). According to a survey of 2,440 patients, celiac disease is primarily diagnosed by gastroenterologists and is rarely diagnosed by primary care physicians [3]. 

A strict gluten-free diet is the only available treatment for CD, and it should only be started after the diagnosis has been firmly established [1]. Failure to treat celiac patients who have recently been diagnosed and have gluten in their diet on time can result in severe complications, including chronic and severe diarrhea, T-cell lymphoma entropy, and colon cancer [8]. One possible cause of long diagnostic delays is a lack of disease knowledge among healthcare providers. Furthermore, patients' adherence to gluten-free diets may be influenced by a lack of knowledge about CD [4]. 

CD complications are also linked to a lack of knowledge about diagnosis and treatment among healthcare professionals. Because the prevalence of atypical presentation in CD patients is higher than expected, and approximately 40% of those diagnosed with CD are adults, healthcare professionals may miss a timely diagnosis of CD. Compared to what the health system expects, internal healthcare professionals' knowledge and skills are insufficient, particularly in the provision of CD diagnosis and treatment services. The training of healthcare professionals is also necessary to improve their knowledge and abilities, in addition to the curriculum revision [9]. Growing evidence points to the disease's underdiagnosis, particularly in non-Western countries that were previously thought to be unaffected [10].

Among Saudi patients, CD is very common [11]. Little is known about healthcare professionals' knowledge of CD among the Saudi population. Therefore, this study was designed to assess the reasons for CD underdiagnosis and the perception of healthcare professionals toward CD.

## Materials and methods

Study design, setting, and participants

A descriptive cross-sectional study recruited gastroenterologists, gastroenterology fellows, internal and family medicine specialists, residents, and general practitioners from the Eastern Province of Saudi Arabia who worked in private or public health centers in various cities of the province during the year 2023, between May and July. The study was approved by the Ethics Committee of King Faisal University (KFU-REC-2023-MAY-ETHICS868, date of approval 18/5/2023). The required sample size was 385 using (n = z2 (p*q)/d2) formula, where the margin of error (E) equals 0.05; 180 out of the 385 randomly selected participants who met the inclusion criteria completed the questionnaire.

Informed consent was obtained from all participants prior to their participation in the study. Consent was provided, explaining the purpose of the study, the voluntary nature of their participation, and any potential risks or benefits associated with their involvement. Participants were informed that their participation was confidential and that they had the right to withdraw from the study at any time without penalty.

Questionnaire development and data collection

A link to a Google form with the questionnaire was distributed for completion. Every participant has responded voluntarily to the 18-item questionnaire. The study used a structured questionnaire developed by Jinga et al. [12]. It was divided into two sections. The first section included questions about the demographic characteristics and professional experience of the physicians, while the second section included questions about the physicians' knowledge and attitudes toward celiac disease. Participants were asked to identify the characteristics, epidemiology, diagnosis and management of CD in order to assess their attitudes and knowledge.

Data analysis

After the data were extracted, they were revised, coded, and fed to the statistical software IBM SPSS version 22 (SPSS, Inc., Chicago, IL). All statistical analysis was done using two-tailed tests. A P value less than 0.05 is considered statistically significant. Regarding celiac disease knowledge and awareness, each correct answer was scored one point, and each incorrect answer was scored zero. The overall knowledge score was obtained by summing up all items into discrete scores, where physicians with an overall score less than 60% of the total were considered to have poor knowledge, while others with an overall score of 60% of the total or more were considered to have a good knowledge level. A descriptive analysis based on frequency and percent distribution was done for all variables, including physicians’ bio-demographic data, specialty, and professional degree. Also, physicians’ knowledge and awareness about celiac disease were tabulated, while their overall knowledge level was graphed. Cross-tabulation was used to assess factors associated with physicians’ knowledge and awareness regarding celiac disease. These factors were tested using the Persons’ chi-square test and the exact probability test for small frequency distributions.

## Results

Characteristics of the participants

Table 1 shows that a total of 180 eligible physicians were included. Physician ages ranged from 20 to 60 years, with a mean age of 26.9 ± 12.7 years and the vast majority (66.7%) aged less than 30 years. Exactly 126 (70%) physicians were male. As for specialty, 72 (40%) were family medicine physicians, 57 (31.7%) were general practitioners, 49 (27.2%) were internal medicine physicians, and only 2 (1.1%) were gastroenterology physicians. A total of 82 (45.6%) were residents, 60 (33.3%) were GPs, 20 (11.1%) were specialists, and 18 (10%) were consultants.

**Table 1:** Sociodemographic characteristics of the participants (N = 180).

Personal data	No	%
Age in years		
<30	120	66.7%
30-40	47	26.1%
41-50	8	4.4%
51-60	5	2.8%
Gender		
Male	126	70.0%
Female	54	30.0%
Specialty		
Family medicine	72	40.0%
Gastroenterology	2	1.1%
General practitioner (GP)	57	31.7%
Internal medicine	49	27.2%
Professional degree		
GP	60	33.3%
Resident/fellow	82	45.6%
Specialist	20	11.1%
Consultant	18	10.0%

Participants’ knowledge and perception

About 49.4% of the study physicians know that adult celiac disease is rare, but only 19.4% frequently recommend celiac disease serology to their patients. Regarding the most frequent antibodies prescribed for CD screening, 92.2% reported for anti-transglutaminase and 23.3% for anti-endomisium. A total of 43.9% of the study physicians frequently recommended intestinal biopsy to patients with positive celiac disease serology. As for situations to recommend celiac disease serology determination, 90.6% know about chronic diarrhea, 81.7% reported weight loss, 61.1% reported abdominal pain of unknown etiology, 46.7% told about unresponsive iron-deficiency anemia, 43.9% told about type I diabetes mellitus, and 39.4% knew about autoimmune thyroiditis, as shown in Table 2.

**Table 2:** Physicians’ knowledge about celiac disease.

Knowledge items		No	%
Do you consider adult celiac disease	Frequent	64	35.6%
	Rare	89	49.4%
	Very rare	19	10.6%
	Don't know	8	4.4%
Did you recommend celiac disease serology to your patients?	Frequent	35	19.4%
	Rare	112	62.2%
	Never	33	18.3%
The most frequent antibodies prescribed for celiac disease screening	Anti-transglutaminase	166	92.2%
	Anti-endomisium	42	23.3%
	Anti-gliadin	26	14.4%
	Anti-deamidated gliadin	15	8.3%
Do you recommend intestinal biopsy to patients with positive celiac disease serology?	Frequent	79	43.9%
	Rare	56	31.1%
	Never	45	25.0%
Do you recommend celiac disease serology determination in the following situations?	Chronic diarrhea	163	90.6%
	Abdominal pain of unknown etiology	110	61.1%
	Weight loss	147	81.7%
	Unresponsive iron-deficiency anemia	84	46.7%
	Type I diabetes mellitus	79	43.9%
	Autoimmune thyroiditis	71	39.4%
	Herpetiforme	43	23.9%
	Osteoporosis	14	7.8%
	Infertility	15	8.3%
	Unexplained transaminases raise	21	11.7%
	Dermatitis	7	3.9%
	Constipation	40	22.2%
	Autoimmune hepatitis	33	18.3%
	Sjögren syndrome	21	11.7%
	Turner syndrome	8	4.4%

Table 3 depicts the risk of CD: 24.4% think that cancer risk in patients with celiac disease is moderate, 23.9% think that lymphoma risk in celiac disease patients is moderate, but 61.7% know that adult celiac disease is a moderately severe pathology, and 47.8% know that it is a moderately disabling disease. About 70% of the study physicians always prescribe a gluten-free diet to patients with celiac disease, and 27.8% recommend an intestinal biopsy for a gluten-free diet. Also, 39.4% always recommend IgA screening for celiac disease patients. A total of 58.9% know that celiac disease diagnosis should be performed in specialized medical centers, while 53.3% said that celiac disease management should be performed in any medical center.

**Table 3:** Physicians’ knowledge about celiac disease, continued.

Knowledge, continued		No	%
The cancer risk in patients with celiac disease is	Low	34	18.9%
	Moderate	44	24.4%
	High	17	9.4%
	Don't know	85	47.2%
The lymphoma risk in celiac disease patients is	Low	21	11.7%
	Moderate	43	23.9%
	High	31	17.2%
	Don’t know	85	47.2%
The adult celiac disease is	Mild pathology	54	30.0%
	A moderate-severe pathology	111	61.7%
	A severe pathology	15	8.3%
The adult celiac disease is	A less debilitating disease	69	38.3%
	A moderately disabling disease	86	47.8%
	A debilitating disease	25	13.9%
Do you prescribe a gluten-free diet in patients with celiac disease?	Always	126	70.0%
	Frequent	25	13.9%
	Rare	16	8.9%
	Never	13	7.2%
Which are the most frequent criteria for which you recommend a gluten-free diet?	Intestinal biopsy	50	27.8%
	Digestive symptoms	38	21.1%
	Positives anti-gliadin antibodies	17	9.4%
	Positives auto-antibodies	75	41.7%
Do you recommend IgA screening in celiac disease patients?	Always	71	39.4%
	Sometimes	85	47.2%
	Never	24	13.3%
Celiac disease diagnosis should be performed in	Specialized medical centers	106	58.9%
	Any medical center	74	41.1%
Celiac disease management should be performed in	Specialized medical centers	96	53.3%
	Any medical center	84	46.7%

**Figure 1:**
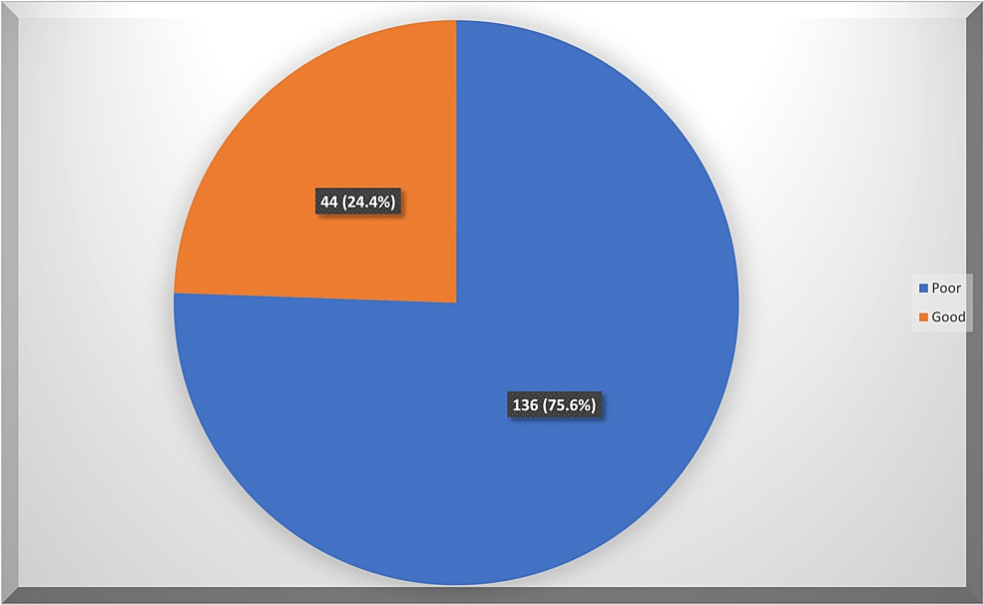
Overall knowledge level regarding celiac disease.

The overall knowledge level regarding celiac disease among study physicians in the Eastern region of Saudi Arabia is shown in Figure 1. Exactly 136 (75.6%) of the study physicians had an overall poor knowledge level regarding CD, and 44 (24.4%) had a good knowledge level.

Table 4 illustrates that 50% of gastroenterologists had an overall good knowledge level regarding CD compared to 14% of GPs, with statistical significance (P = 0.001). Also, 45.1% of internal medicine physicians had good knowledge compared to 16.3% of other specialties (P = 0.001). Other factors showed an insignificant association with physicians’ knowledge levels.

**Table 4:** Factors associated with physicians’ knowledge regarding celiac disease. P: Pearson X^2^ test, $: exact probability test, *P<0.05 (significant), GE: gastroenterology.

Factors	Overall knowledge level				p-value
	Poor		Good		
	No	%	No	%	
Age in years					0.523^$^
<30	93	77.5%	27	22.5%	
30-40	33	70.2%	14	29.8%	
41-50	7	87.5%	1	12.5%	
51-60	3	60.0%	2	40.0%	
Gender					0.940
Male	95	75.4%	31	24.6%	
Female	41	75.9%	13	24.1%	
Specialty					0.001*^$^
Family medicine	59	81.9%	13	18.1%	
Gastroenterology	1	50.0%	1	50.0%	
General practitioner (GP)	49	86.0%	8	14.0%	
Internal medicine	27	55.1%	22	44.9%	
Specialty					0.001*
Medicine/GE	28	54.9%	23	45.1%	
Other specialty	108	83.7%	21	16.3%	
Professional degree					0.188^$^
GP	51	85.0%	9	15.0%	
Resident/fellow	59	72.0%	23	28.0%	
Specialist	13	65.0%	7	35.0%	
Consultant	13	72.2%	5	27.8%	

## Discussion

Celiac disease is a genetic autoimmune disease that impairs the absorption of nutrients from food by damaging the villi of the small intestine [13]. Approximately one in 133 Americans, or 1% of the population, has celiac disease [2,3]. However, current screening studies indicate a potentially higher prevalence of the disease in the United States [2]. Symptoms of celiac disease include digestive symptoms like bloating, abdominal pain, and diarrhea, as well as symptoms unrelated to digestion, like iron-deficiency anemia and osteoporosis [14,15]. Treatment for celiac disease involves maintaining a gluten-free diet, which can alleviate symptoms and reverse intestinal damage. Raising awareness about celiac disease is crucial in promoting early diagnosis and management. Greater education about the condition can also break down stigmas and myths surrounding a gluten-free diet, which is often the primary treatment for celiac disease [16,17].

The current study aimed to assess physicians' awareness and knowledge regarding CD, which may affect their diagnostic ability and management. The study revealed that only one-fourth of the physicians were knowledgeable regarding CD. In more detail, about half of the physicians know that adult celiac disease is rare, but only one-fifth frequently recommend celiac disease serology to their patients. Regarding the most frequent antibodies prescribed for celiac disease screening, most of the participants reported anti-transglutaminase and also anti-endomisium. In less than half of the study, physicians frequently recommended intestinal biopsy to patients with positive celiac disease serology. As for situations to recommend celiac disease serology determination, most of them know about chronic diarrhea; 81.7% reported weight loss; less than two-thirds reported abdominal pain of unknown etiology; less than half told about unresponsive iron-deficiency anemia; type I diabetes mellitus; and more than one-third knew about autoimmune thyroiditis. As for celiac disease among adults, the Eastern region, Saudi Arabia, continued. As for the risk of CD, 24.4% think that cancer risk in patients with celiac disease is moderate, about one-fourth think that lymphoma risk in celiac disease patients is moderate, but about two-thirds know that adult celiac disease is a moderately severe pathology, and nearly half of them know that it is a moderately disabling disease.

More than half of the physicians know that celiac disease diagnosis should be performed in specialized medical centers and also told that celiac disease management should be performed in any medical center. Knowledge was significantly higher among gastroenterologists and internal medicine physicians than others. Zipser et al. reported that nearly all physicians (95%) knew of wheat intolerance, but only one-third knew that the onset of symptoms in adulthood is common [3]. As for symptoms, the most known for physicians were diarrhea, but fewer knew of the common symptoms of irritable bowel syndrome (71%), chronic abdominal pain (67%), fatigue (54%), depression and irritability (24%) or associations with diabetes (13%), anemia (45%) or osteoporosis (45%), or diagnosis by endomysial antibody tests (44%). Also, Kozhakhmetova et al. revealed poor awareness in 59.4% of physicians associated with work in republican, provincial, district, rural/village hospitals [18]. On the other hand, Assiri et al. in Saudi Arabia documented higher awareness regarding CD among healthcare professionals, where about 80% of them had good knowledge [19]. Interns and residents had fair to good knowledge, but registrars, specialists, and even the consultants were less knowledgeable of celiac disease. Sahin et al. in Turkey found that most of the physicians were general practitioners (37.1%). The highest score in all categories was reported among pediatric gastroenterologists. There were significant differences between the four groups of healthcare providers in terms of the subsections of the overall mean score.

Gaps in knowledge about celiac disease among doctors and other healthcare providers are estimated in two new European studies [12]. In the first, the results of 1,400 physicians from five central European countries showed that knowledge regarding celiac disease was poor. On average, only half of the questions were correctly answered by physicians, including general practitioners, pediatricians, and pediatric and adult gastroenterologists. The second study, done by Polish researchers, found that healthcare providers largely don’t realize that people with celiac disease on a gluten-free diet are still at risk for nutritional deficiencies. Nearly 50 percent of the more than 400 doctors, nurses, dietitians, and medical students surveyed said people with treated celiac disease are not at risk of nutritional deficiencies.

Strength and limitations

This is the first study that has investigated the knowledge and attitudes of physicians toward celiac disease in the Eastern Province of Saudi Arabia and their role in the underdiagnosis of the disease among the adult population. However, due to the small size of the completed responses, the results cannot be generalized. 

## Conclusions

The study found that a majority of physicians in the Eastern region of Saudi Arabia had poor knowledge about celiac disease (CD). Only 24.4% of physicians had good knowledge about CD, while 75.6% had poor knowledge. This lack of knowledge was seen across all specialties; however, it was more pronounced among general practitioners (GPs) and family medicine physicians. There were several factors that are associated with physicians' knowledge about CD. Physicians who were more experienced were more likely to have good knowledge about CD. Additionally, physicians who worked in specialized medical centers were more likely to have good knowledge about CD than those who worked in general medical centers. The study's findings suggest that there is a need to improve physicians' knowledge about CD in Saudi Arabia. This could be done through targeted educational interventions, such as workshops or online courses. Additionally, it is important to ensure that physicians have access to up-to-date information about CD, such as medical textbooks and journals. The study's findings also have implications for patients with CD. Patients who are diagnosed by physicians with poor knowledge about CD may not receive the best possible care. This could lead to delays in diagnosis, inadequate treatment, and an increased risk of complications. Overall, the study's findings highlight the need to improve physicians' knowledge about CD in Saudi Arabia. This is important for both patients and physicians, as it can lead to better diagnosis and treatment of CD.
